# Linkage to HIV Care and Early Retention in Care Rates in the Universal Test-and-Treat Era: A Population-based Prospective Study in KwaZulu-Natal, South Africa

**DOI:** 10.1007/s10461-022-03844-w

**Published:** 2022-09-13

**Authors:** Edward Nicol, Wisdom Basera, Ferdinand C Mukumbang, Mireille Cheyip, Simangele Mthethwa, Carl Lombard, Ngcwalisa Jama, Desiree Pass, Ria Laubscher, Debbie Bradshaw

**Affiliations:** 1 Burden of Disease Research Unit, South African Medical Research Council, Tygerberg, 7505, Cape Town, P.O. Box 19070, South Africa; 2 Division of Health Systems and Public Health, Stellenbosch University, Cape Town, South Africa; 3 School of Public Health and Family Medicine, University of Cape Town, Cape Town, South Africa; 4 School of Public Health, University of the Western Cape, Bellville, South Africa; 5 Centers for Disease Control and Prevention, Pretoria, South Africa; 6 Biostatistics, South African Medical Research Council, Cape Town, South Africa

**Keywords:** HIV care continuum, Linkage to care, HIV prevention, Retention, HIV epidemiology, Universal Test and Treat, South Africa

## Abstract

HIV linkage, and retention are key weaknesses in South Africa’s national antiretroviral therapy (ART) program, with the greatest loss of patients in the HIV treatment pathway occurring before ART initiation. This study investigated linkage-to and early-retention-in-care (LTRIC) rates among adults newly diagnosed with HIV in a high-HIV prevalent rural district. We conducted an observational prospective cohort study to investigate LTRIC rates for adults with a new HIV diagnosis in South Africa. Patient-level survey and clinical data were collected using a one-stage-cluster design from 18 healthcare facilities and triangulated between HIV and laboratory databases and registered deaths from Department of Home Affairs. We used Chi-square tests to assess associations between categorical variables, and results were stratified by HIV status, sex, and age. Of the 5,637 participants recruited, 21.2% had confirmed HIV, of which 70.9% were women, and 46.5% were aged 25–34 years. Although 82.7% of participants were linked-to-care within 3 months, only 46.1% remained-in-care 12 months after initiating ART and 5.2% were deceased. While a significantly higher proportion of men were linked-to-care at 3 months compared to women, a significant proportion of women (49.5%) remained-in-care at 12 months than men (38.0%). Post-secondary education and child support grants were significantly associated with retention. We found high linkage-to-care rates, but less than 50% of participants remained-in-care at 12 months. Significant effort is required to retain people living with HIV in care, especially during the first year after ART initiation. Our findings suggest that interventions could target men to encourage HIV testing.

## Introduction

In 2014, The Joint United Nations Program on HIV/AIDS (UNAIDS) launched the ’90-90-90’ goal ensuring that by 2020, 90% of people living with HIV (PLHIV) are diagnosed, 90% of those diagnosed are initiated on antiretroviral therapy (ART), and 90% of those on ART are virally suppressed [1]. The World Health Organization (WHO) recommended the ‘Universal Test-and-Treat’ (UTT) strategy – initiating all individuals testing positive on ART irrespective of their CD4 count and clinical staging [2] – as a fast-track strategy to achieve this goal. UTT is defined as starting ART within 14 days of an initial HIV-positive diagnosis. In May 2016, following the recommendations by WHO, the South African government announced the phased rollout of the UTT strategy from September 2016 [3].

An estimated 7.9million South Africans were living with HIV in 2017 [4], with an incidence of 276,000 in the same year [5]. It is also estimated that by 2017, 84.9% of South Africans knew their HIV status. Of these, 70% were on ART and of those on ART, 87.3% had achieved viral suppression (latest viral load < 400 copies/cm^3^) [4]. It is predicted that through the UTT program, South Africa could end HIV as a public health concern by 2030. Nevertheless, the evidence on the impact of UTT and how UTT has improved the efficiency and quality of HIV care at the population-level is still scarce [6]. Despite the drop in HIV incidence (–5.7%) and HIV-related deaths (–10.5%) from 2007 to 2017, there is a dearth of evidence to inform the impact of UTT toward the 90-90-90 goal by 2020 [5]. However, the general impression was that South Africa is making progress in the fight against the HIV epidemic.

The literature suggests that ending AIDS by 2030 depends on how successful health systems are in linking PLHIV testing HIV-positive into care, completion of a first medical clinic visit after HIV diagnosis, and achieving early and lifelong retention in care [7]. Before the UTT era, only 45% of newly diagnosed HIV-positive individuals were linked to care in the KwaZulu-Natal Province of South Africa [8], with an estimated 79% considered late presentation (i.e., HIV diagnosis was done when the CD4 + cell count had dropped to ≤ 500 cells/µL and/or who had clinical manifestations of AIDS at the time of diagnosis) for HIV care [9]. It was also estimated that the retention in care rates of PLHIV in low- and middle-income countries before UTT at 12 months was 78% [10]. While the linkage to and retention in care rates of PLHIV before the UTT era was well-documented, little is known on these rates in the era of UTT. This study aimed to investigate the linkage to and retention in care proportions of an HIV-positive cohort in a high HIV prevalent rural district in South Africa.

## Methods

### Study Framework

**Figure 1:**
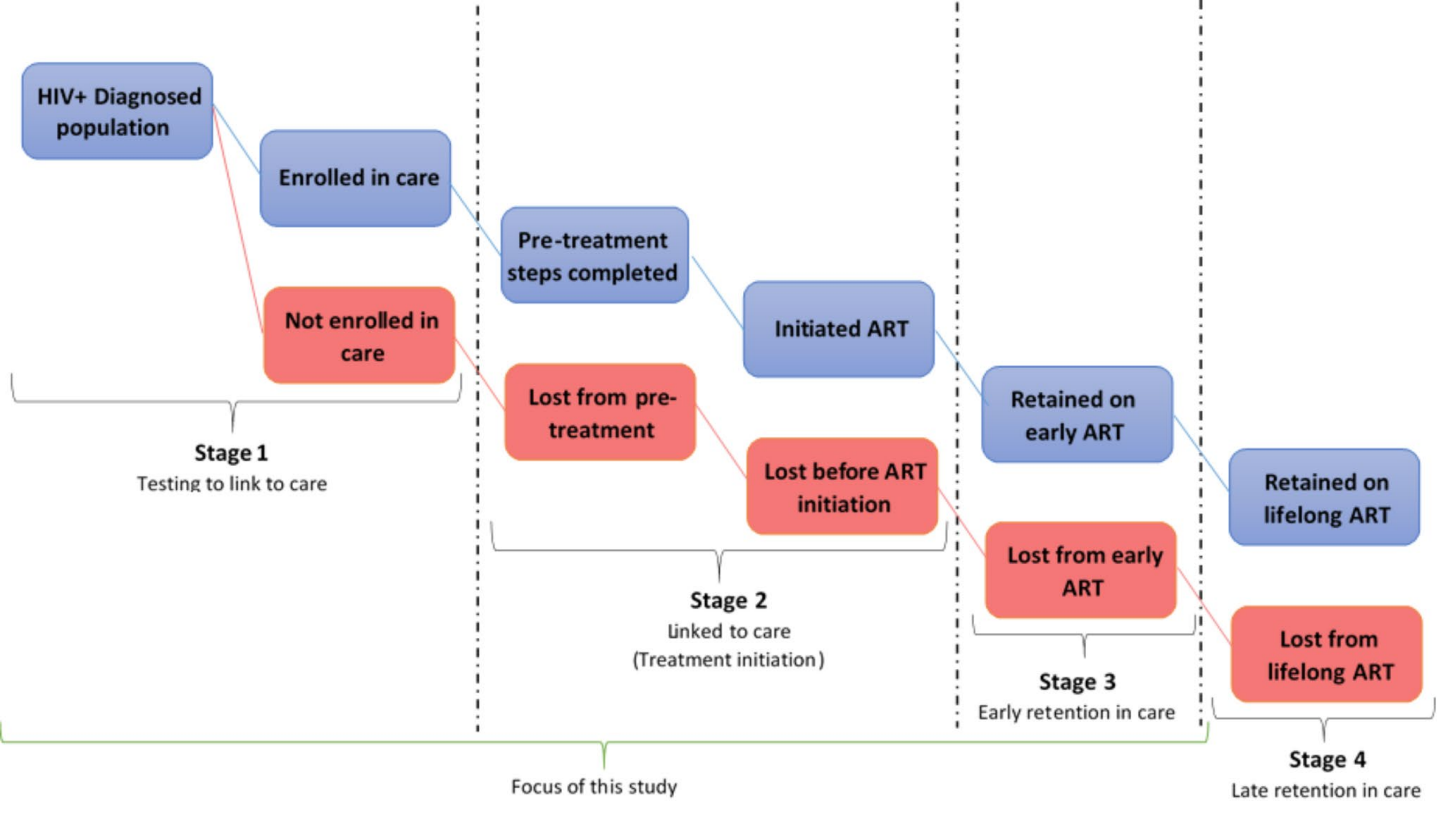
A new 4-stage HIV Care Cascade and parallel loss-to-care.

This study was guided by the new cascade of HIV care in the UTT era (Fig.1) [11]. We found this cascade useful because it provides both breadth (ranging from first to final event) and depth (number of cascade stages that was analysed) [12], which aligned with our study objectives.

Even though this new cascade of UTT is depicted to be linear in nature, in reality, patients cycle in and out of care, leaving at one stage and re-entering at another in what has been described as the “side door” in the cascade [11]. Our study focused on the first three stages of the new HIV treatment cascade.

### Study Design

An observational cohort design was undertaken in a single high-prevalence rural district over 21 months from December 2017 to August 2019, in 18 primary health care facilities in the uThukela district. Adults older than 18 years of age seeking testing for HIV in different facilities were approached to enroll and requested to complete a self-administered questionnaire.

### Setting

**Figure 2:**
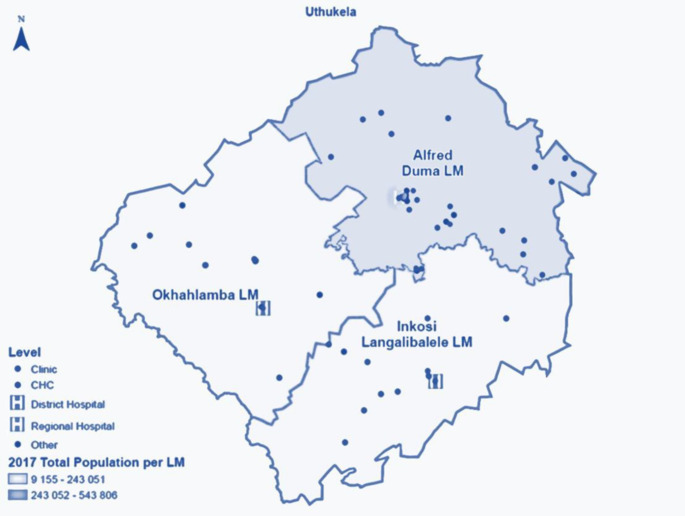
Map of the uThukela rural district for the Linkage to Care study in uThukela district between December 2017 – August 2019.

The district is comprised of three newly formed local municipalities (LMs) – Fig.2. The Alfred Duma LM is the most populated (51%) with the largest town (Ladysmith), which is also the seat of power for both the Alfred Duma local municipality (LM) and the uThukela district municipality (DM). The Inkosi Langalibalele LM is the second most populated (30%), with a sparsely rural and densely urban population. The Okhahlamba LM has the smallest population (19%) with the largest number of service delivery challenges, primarily due to remote mountainous areas, poor road infrastructure, and the lowest ratio of fixed clinics.

### Sampling approach

We undertook a sample size calculation based on the primary outcome; the proportion linked to HIV care. Linkage to care for enrolled HIV-positive adult participants was defined as the completion of a first medical clinic visit within 3 months of HIV-positive diagnosis as evidenced by a record in Tier.Net. Data on linkage to HIV care rates and the uptake of UTT in uThukela district was unknown at the time of designing the study. However, findings of previous surveillance data from KwaZulu-Natal in South Africa demonstrate an average linkage to care of 62% post-HIV testing in the first year [8].

We, therefore, proposed a linkage to care rate of 10% higher than 62% based on the possible impact of UTT on HIV care uptake rates. Assuming a null proportion of 62% (i.e., the reported linkage to care proportion from previous systematic reviews) and a proposed proportion of 72% (based on the potential UTT influence), a minimum sample size of 18 clusters with a cluster size of 50 participants was required to test the difference between the linkage to care proportions with 80% power (Table1**)**. We assumed cluster randomization with an interclass correlation (ICC) (of the clusters in consideration) of 0.02 and significance level of 0.05 [13]. The sample and power calculations were done using Stata 16.0 [14].

**Table 1:** Power and sample size calculations using the coefficient of variation between clusters in the Linkage to Care study in uThukela district between December 2017 – August 2019

Matched pairs										
p_1_	p_2_	delta	alpha	Beta	k_1_	k_2_	m_1_	m_2_	rho	N
0.62	0.72	0.10	0.05	0.80	9	9	50	50	0.02	900

Where *p - linkage to care proportions; delta – difference between proportions; alpha - z values used for calculating type 1 error; beta - z value used for calculating power; rho – inter-cluster correlation; m – average cluster size and k - number of clusters. N - sample size for study period allowing for cluster randomization*

**Figure 3:**
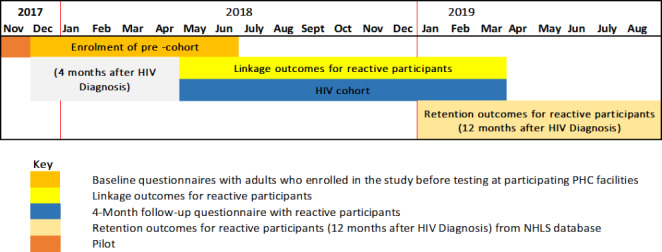
Schematic overview of the data collection process for the Linkage to Care study in uThukela district (2017–2019).

We selected 18 facilities from the district based on the HIV testing uptake rates; these were facilities likely to yield the required minimum number of tests to increase the probability of enrolling 10 people per day. We assumed a conservative number of 10 people to be enrolled from the targeted 18 facilities per month equaling to 60 participants over the six-month data collection period yielding a possible 1,080 participants, which would cover the required sample size of 900 participants (Table1). The study adopted a convenience sampling of reactive participants until this target was reached from the participating facilities (Fig.3). There was a pilot study conducted in November 2017 over two weeks to test the data collection instruments and review the study processes. Over the next few months (December 2017 to June 2018) the baseline cohort of HIV reactive participants was enrolled using the study inclusion criteria.

Participants with a positive result for antibodies against HIV were regarded as reactive participants. The linking of the enrolled participants was assessed four months after the date of enrolment via a follow-up interview. Beyond the four-month mark until 12 months, the linkage outcomes i.e., continued visitation by the reactive participants to the health care system was assessed. At 12 months, retention of the reactive participants was then reviewed using data from the NHLS database and the rapid mortality survey (RMS), which uses the participants’ national identification number and contains information on the deaths registered by the South African Department of Home Affairs (Fig.3).

On the day of enrolment, trained fieldworkers had a waiting room talk which informed people attending the clinic about the research and invited them to volunteer to participate. Individuals who were willing to volunteer were given the study information and screened for enrollment in a private room. Eligible participants were given full study information, completed the informed consent process, and were then enrolled in the study. Participants consented to having research staff obtain their HIV test outcomes from the clinic records, access records in health care databases, and track outcomes including vital status.

To be eligible for enrolment in the study, participants must have been aged 18 years and older, intended to take an HIV test in one of the participating primary care facilities from December 2017 to June 2018 and have had access to a cell phone and willingness to provide contact details. Participants who were under age 18 years at the time of enrolment, without access to a cell phone or unwilling to provide contact details, and those testing at non-medical sites – prison health facilities or through antenatal care, were excluded from the study.

### Data collection tool and process

Data were collected using isiZulu and English REDCap-based questionnaires, and medical record reviews. While prospective participants were waiting in the HIV testing queue, fieldworkers invited them to enroll in the study. Each questionnaire took between 45 and 60min to complete, depending on whether it was self-administered or completed with assistance. Demographic data and potential barriers and enablers of linkage to care information were collected at recruitment and during the 4-month follow-up visit. The data collected using the questionnaire included demographic information, socio-economic characteristics, reasons for testing, and intimate partner violence. At the end of the day, each participant’s HIV results were retrieved from clinic records in order to categorize them for the study.

Tier.Net, an electronic database for Infectious Disease Epidemiology and Research [15] and the National Health Laboratory System’s (NHLS) TrakCare database, also referred to as the NHLS’ central data warehouse (CDW) were used to track participants who tested positive as they interacted with the health care system for CD4, viral load measurements, and ART use. The NHLS’ central data warehouse (CDW) probabilistic linkage algorithm was used to link the results for individuals using names, dates of birth, sex, initial laboratory identification, testing facility name etc.

The vital status of participants was checked at the end of the 12 months follow-up to explain their possible non-participation – linkage to care and retention in care. This was done using the RMS. As for retention in care, the presence and timing of the most recent viral load measurements for each patient obtained from TIER.Net/NHLS was considered [16].

Once extracted from the NHLS’ CDW, TIER.Net and the RMS, the records were de-identified and merged with the REDCap-based participant questionnaires using the unique enrolment ID number in Stata 16.0 [14] for analysis. Linkage to HIV care was defined as successful completion of a first medical clinic visit within 3 months of HIV-positive diagnosis as evidenced by a record in Tier.Net [17, 18]. Retention in care was defined as remaining in contact with HIV care services once linked to the services viz. ART initiation and the frequency of clinic visits or recorded CD4 count and viral load tests conducted within one year and captured in Tier.Net and the NHLS database [19].

### Data analysis

Questionnaire data were collected electronically via REDCap [20], while mortality data and patient blood test results were extracted from the RMS and NHLS CDW respectively using Microsoft Excel. Statistical analyses were completed using the svyset command in STATA 16.0 (14) to incorporate the one stage cluster study design of the sample. The primary sampling unit was the name of the facility which represented the cluster were the survey participants came from stratified by the facility type. There was no finite population correction since the number of possible participants was not known beforehand. Once set, proportions were reported with their respective 95% confidence intervals (CIs) since most of the variables were categorical. Continuous data were reported as medians, interquartile range (IQR) since the data was skewed. Linkage to and retention in care were expressed as proportions of the HIV-positive cohort. The Mann-Whitney test was used to investigate differences between numerical variables, while the Pearson’s chi-squared test which was used to assess associations between categorical variables was corrected using the second order correction of Rao and Scott and converted into an F statistic [21]. Confidence intervals and p-values of less than or equal to 0.05 were reported to consider statistical significance for the various characteristics of the sample.

## Results

**Figure 4:**
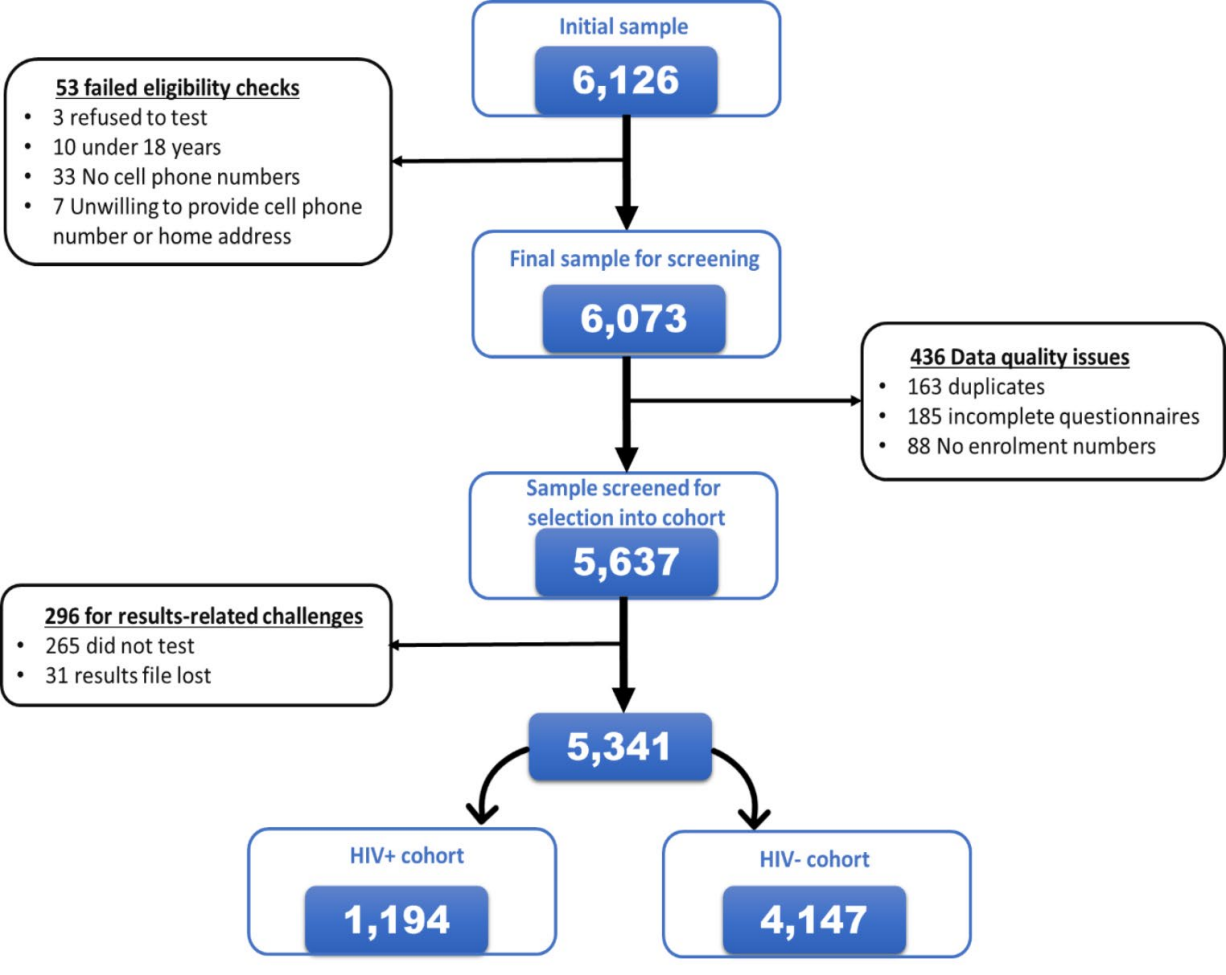
Consort diagram detailing the recruitment of the study participants into the Linkage to Care study in uThukela district between December 2017 – June 2018.

**Figure 5:**
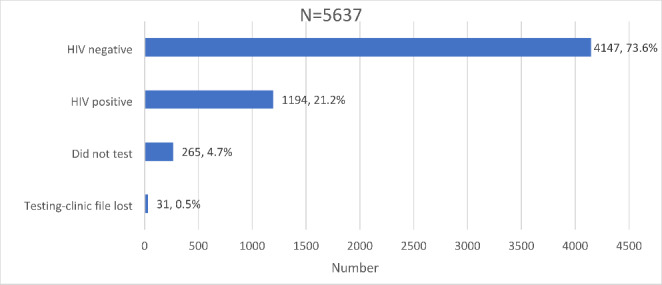
Distribution of HIV testing outcomes and awareness in the Linkage to Care study cohort in uThukela district between December 2017 - August 2019.

A total of 5,637 participants were recruited from December 2017 to June 2018 after screening 6,126 participants at enrolment for inclusion eligibility. A small proportion of participants were excluded from the study sample because of failed eligibility checks (0.9% – 53/6,126) and data quality issues (7.1% – 436/6,126) (Fig.4). Participants were grouped into two cohorts for analysis: newly diagnosed HIV-positive and those who tested negative (Fig.5).

### HIV testing and status awareness

Those who underwent HIV testing as a proportion of the screened study sample was 95.3% (5,372/5,637). A small proportion, 0.6% (31/5,372) of those who tested on the day of recruitment had their records misplaced or lost at the facility and 4.7% (265/5,637) of the participants included those who intended to test but did not test on the day of recruitment. Overall, the proportion of those who did not receive their results was 5.3% (296/5,637) inclusive of those who did not test and those who had their results lost.

Of the proportion that knew their status, 94.7% (5,341/5,637) had the following outcomes for their HIV status; 73.6% (4,147/5,637) tested HIV negative while 21.2% (1,194/5,637) tested HIV-positive (Fig.5).

The majority of participants who tested were women – 69.6% (3,921/5,637) and had either a high school or a tertiary education 92.0% [(2,866 + 2,257)/5,566]. Overall, the association between socio-demographic characteristics and knowledge of HIV status after recruitment into the study was not statistically significant for most variables (Table2).

**Table 2:** Demographic characteristics of participants disaggregated by knowledge of HIV status on the day of recruitment into the Linkage to Care study in uThukela district between December 2017 - June 2018

*Variable*	*Total (N = 5,637)*			*Knows HIV status (KHS) (N = 5,341)*			*Doesn’t know HIV status (DKHS) (N = 296)*				
	***n***	***%***	***95% CI***	***n***	***%***	***95% CI***	***n***	***%***	***95% CI***	**F value**	**p-value**
**Nationality** South African citizen Other SADC Other African Other outside Africa None	5,572 51 8 4 2	98.8 0.9 0.1 0.1 0	97.8–99.4 0.4–2.2 0.07–0.3 0.02–0.2 0.01–0.1	5,279 49 7 4 2	98.8 0.9 0.1 0.1 0	97.8–99.4 0.4–2.2 0.1–0.3 0.02–0.23 0.01–0.15	293 2 1 0 0	99.0 0.7 0.3 0 0	97.2–99.6 0.1–3.7 0.1–1.5 - -	0.3	0.78
**Facility type** Clinics Gateway Hospital Mobile clinic	3,772 870 300 695	66.9 15.4 5.3 12.3	58.1–74.7 9.4–24.4 2.6–10.6 9.8–15.4	3,558 860 296 627	66.6 16.1 5.5 11.7	57.8–74.4 9.9–25.2 2.6–11.3 9.6–14.3	214 10 4 68	72.3 3.4 1.4 23.0	38.6–91.6 0.6–16.7 0.1–11.9 6.1–57.6	1.9	0.17
**Sex** Male Female	1,716 3,921	30.4 69.6	27.4–33.7 66.3–72.6	1,648 3,693	30.9 69.1	27.9–33.9 66.1–72.1	68 228	23.0 77.0	16.4–31.3 68.7–83.7	4.8	**0.05**
**Ethnicity** Black African Colored/Mixed ancestry White Indian/Asian	5,619 12 1 5	99.7 0.2 0.02 0.1	99.2–99.9 0.07–0.6 0.002-0.2 0.04–0.2	5,325 10 1 5	99.7 0.2 0.02 0.1	99.2–99.9 0.1–0.6 0.002-0.2 0.04–0.2	294 2 0 0	99.3 0.7 0 0	95.0-99.9 0.1–5.1 - -	0.9	0.44
**Education level** No education Primary education High school education Tertiary education	107/5,566 336/5,566 2,866/5,566 2,257/5,566	1.9 6.0 51.5 40.5	1.4–2.7 5.0-7.3 47.8–55.2 36.5–44.7	102 315 2,717 2,144	1.9 6.0 51.5 40.6	1.4–2.7 5.0-7.2 47.6–55.3 36.4–45.0	5 21 149 113	1.7 7.3 51.7 39.2	0.6–5.1 3.7–13.8 44.6–58.8 31.5–47.6	0.2	0.84
**Age, median (IQR)**	5,627	28	23–35	5,333	28	23–35	294	26	22–33	-2.6*	**0.01**
**Age categories** 18–24 years 25–29 years 30–34 years 35–49 years 50 + years	1,943/5,627 1,310/5,627 916/5,627 1,029/5,627 429/5,627	34.5 23.3 16.3 18.3 7.6	29.8–39.6 21.7–25.0 14.2–18.6 15.7–21.2 6.0-9.6	1,824 1,243 875 984 407	34.2 23.3 16.4 18.5 7.6	29.4–39.4 21.7–25.0 14.3–18.8 15.9–21.3 6.0-9.7	119 67 41 45 22	40.5 22.8 13.9 15.3 7.5	33.2–48.2 18.8–27.4 9.9–19.4 10.1–22.6 4.5–12.1	1.2	0.32
**Marital status** Married (living together) Married (living separately) Cohabiting Dating Single	475/5,517 178/5,517 724/5,517 3,479/5,517 661/5,517	8.6 3.2 13.1 63.1 12.0	6.7–11.0 2.2–4.7 9.8–17.3 56.9–68.8 8.7–16.2	447 161 690 3,305 629	8.5 3.1 13.2 63.2 12.0	6.6–11.0 2.1–4.5 9.8–17.6 56.8–69.1 8.7–16.4	28 17 34 174 32	9.8 6.0 11.9 61.1 11.2	5.5–16.9 3.8–9.3 8.9–15.8 54.9–66.9 6.3–19.1	1.5	0.24
**Access to US$14 in emergencies** Very/somewhat difficult Fairly/very easy	3,412/5,525 2,113/5,525	61.8 38.2	53.2–67.8 32.2–44.7	3,238 2,003	61.8 38.2	55.2–67.9 32.1–44.8	174 110	61.3 38.7	52.8–69.1 30.9–47.2	0.02	0.89
**Received child support grant**	2,537/5,511	46.0	42.0-50.1	2,396	45.8	42.0-49.7	141	50.0	34.6–65.4	0.3	0.56

p-value of$\le 0.05$ was considered statistically significant

p-values derived using Mann Whitney U-test for continuous data

p-values derived using Pearson’s Chi-squared test considered the one stage cluster design

proportions (%) for the columns reported as n/N (except if data is missing - denominator added in the n column) and the associated 95% CI

IQR – Interquartile range; CI – Confidence Interval; US$ – United States of American Dollar; DKHS – Don’t know HIV status; KHS – Knows HIV status

* z value reported

A higher proportion of males knew their HIV status compared to females (96.0%: 1,648/1,716 vs. 94.2%: 3,693/3,921, respectively) which was statistically significant (F = 4.8 and p = 0.05). Those who learned of their status during their visit to the facility were significantly older than those who did not get to know their status (28 years; IQR: 23–35 vs. 26 years; IQR: 22–33 respectively – z=-2.6 and p = 0.01).

### Linked to care

Of the 1,194 participants diagnosed HIV-positive at baseline, 728 (61.0%) and 987 (82.7%) were linked to care within 14 days and 3 months respectively, while 207 (17.3%) were identified as not linked to care at the time. Of those that were linked to care within 3 months after knowing their HIV status and initiating ART, 64.1% (633/987) returned to the facility at the time they were told to do so by the counselor. Reasons given for delaying the return or not returning to the clinic for HIV care during the four-month follow-up period included a lack of money for transportation (26.5%, 236/889), not being able to take time off work (28.9%, 254/879), or inconvenient appointment date (19.6%, 174/888). Also reported were issues with accessing the facilities as they were too far (29.1%, 259/889).

Of the 1,194 HIV-positive participants, 851 (71.3%) were females, 793 (66.4%) came from clinics and 105 (8.8%) were from hospitals. Of the 1,177 participants with their level of education recorded, 665 (56.5%) had high school education while 434 (36.9%) had a tertiary education. Overall, almost all the socio-demographic characteristics were similar with respect to whether participants were linked to care or not, excluding sex (Table3). A higher proportion of males were linked to care at 3 months post HIV-positive diagnosis when compared to females (85.1%: 292/343 vs. 81.7%: 695/851; F = 5.9 and p = 0.03).

**Table 3:** Socio-Demographic characteristics of the HIV-positive participants at baseline and linkage in care at 3 months of follow-up in uThukela district between December 2017 - August 2019 (N = 1,194)

*Variable*	*Total* *(N = 1,194)*			*Linked in care (LiC)* *(N = 987)*			*Not linked in care (nLiC) N = 207)*				
	***n***	***%***	***95% CI***	***n***	***%***	***95% CI***	***n***	***%***	***95% CI***	**F-value**	**p-value**
**Nationality** South African citizen Other SADC Other African None	1,180 11 2 1	98.8 0.9 0.2 0.1	97.1–99.5 0.3–2.6 0.04–0.7 0.01–0.7	976 9 1 1	98.9 0.9 0.1 0.1	97.3–99.5 0.4–2.3 0.01–0.9 0.01–0.9	204 2 1 0	98.6 1.0 0.5 0	94.2–99.7 0.1–6.5 0.1-4.0 -	0.6	0.57
**Facility type** Clinics Gateway Hospital Mobile clinic	793 229 105 67	66.4 19.2 8.8 5.6	54.3–76.7 13.4–26.7 2.4–27.1 3.2–9.7	637 190 100 60	64.5 19.3 10.1 6.1	51.8–75.5 13.1–27.3 3.0-29.5 3.3–11.0	156 39 5 7	75.4 18.8 2.4 3.4	59.5–86.5 9.5–33.8 0.3–18.0 2.0-5.6	3.1	0.07
**Sex** Male Female	343 851	28.7 71.3	25.9–31.7 68.3–74.1	292 695	29.6 70.4	26.9–32.4 67.6–73.1	51 156	24.6 75.4	20.1–29.8 70.2–79.9	5.9	**0.03**
**Ethnicity** Black African Colored/Mixed ancestry Indian/Asian	1,191 2 1	99.7 0.2 0.1	99.2–99.9 0.04–0.7 0.01–0.7	984 2 1	99.7 0.2 0.1	99.0-99.9 0.1–0.8 0.01–0.9	207 0 0	100 0 0	- - -	0.3	0.70
**Education level** No education Primary education High school education Tertiary education	21/1,177 57/1,177 665/1,177 434/1,177	1.8 4.8 56.5 36.9	1.0-3.3 4.0-5.8 50.1–62.7 30.1–44.2	18 50 552 353	1.8 5.1 56.7 36.3	1.1–3.2 4.2–6.3 49.7–63.5 29.2–44.0	3 7 113 81	1.5 3.4 55.4 39.7	0.3–7.4 1.7–6.6 49.1–61.6 32.0–48.0	0.6	0.59
**Age, median (IQR)**	1,193	30	25–37	986	30	25.0–37.0	207	31	25–38	0.3*	0.75
**Age categories** 18–24 years 25–29 years 30–34 years 35–49 years 50 + years	269/1,193 293/1,193 251/1,193 317/1,193 63/1,193	22.5 24.6 21.0 26.6 5.3	19.3–26.2 21.7–27.7 18.7–23.6 23.0-30.5 4.2–6.7	220 243 210 259 54	22.3 24.6 21.3 26.3 5.5	18.7–26.4 21.5–28.1 18.6–24.3 22.7–30.2 4.2–7.1	49 50 41 58 9	23.7 24.2 19.8 28.0 4.3	19.6–28.3 19.1–30.1 17.0–23.0 21.2–36.0 2.4–7.6	0.4	0.76
**Marital status** Married (living together) Married (living separately) Cohabiting Dating Single	68/1,168 29/1,168 178/1,168 725/1,168 168/1,168	5.8 2.5 15.2 62.1 14.4	3.7–9.1 1.4–4.3 11.1–20.6 56.4–67.4 9.4–21.4	55 25 150 593 145	5.7 2.6 15.5 61.3 15.0	3.5-9.0 1.6–4.2 11.0-21.4 55.7–66.5 10.2–21.6	13 4 28 132 23	6.5 2.0 14.0 66.0 11.5	3.3–12.3 0.5–7.7 8.1–23.2 55.5–75.1 5.1–24.0	0.5	0.67
**Access to US$14 in emergencies** Very/somewhat difficult Fairly/very easy	714/1,171 457/1,171	61.0 39.0	52.2–69.1 30.9–47.9	595 378	61.2 38.8	51.9–69.7 30.3–48.2	119 79	60.1 39.9	51.3–68.3 31.7–48.7	0.1	0.74
**Received child support grant**	527/1,169	45.1	42.1–48.1	438	45.2	41.6–48.9	89	44.5	36.0-53.3	0.02	0.89

p-value of$\le 0.05$ was considered statistically significant

p-values derived using Mann Whitney U-test for continuous data

p-values derived using Chi-squared test considered the one stage cluster design

proportions (%) for the columns reported as n/N (except if data is missing - denominator added in the n column) and the associated 95% CI

IQR – Interquartile range; CI – Confidence Interval; US$ – United States of American Dollar; LiC – Linked in Care; nLiC – Not linked in care

* z value reported

### Retention in care

Of the 1,194 individuals who tested HIV-positive, 551 (46.1%) were still accessing care in a health care facility at 12 months. There were some observed differences in the baseline socio-demographic characteristics between participants who remained in care at 12 months and those who dropped out of care (Table4). The proportion of females that remained in care at 12 months was significantly higher than males [49.5%, 419/847 vs. 38.0%, 132/347; p = 0.01). The tertiary education group had the highest proportion of those retained in care at 12 months (51.3%, 215/419) and the least proportion was recorded in the no education group (23.8%, 5/21). Those seeking care in urban areas were more likely to remain in care than those in the rural areas, however, place of residence was not a determining factor for retention in care (F = 2.0 and p = 0.18). In addition, participants who use public transport to access health facilities were more likely to be in care than those who access health facilities by foot (F = 1.0 and p = 0.38). Participants who had access to a child support grant (50.7%, 269/531) were more likely to be retained in care at 12 months post HIV diagnosis (F = 5.8 and p = 0.03). Also, those who found it very difficult to access R200 ($14) in emergency cases 49.5% (348/703) were less likely to be retained in care at 12 months post HIV diagnosis; however, the association was not statistically significant (F = 1.0 and p = 0.34).

**Table 4:** Socio-Demographic characteristics of HIV-positive participants at baseline and retention in care after 12 months of follow-up in uThukela district between December 2017 - August 2019 (N = 1,194)

Variable	Total *(N = 1,194)*			Retained in care (RiC) *(N = 551)*			Not retained in care (nRiC) *(N = 643)*				
	***n***	***%***	***95% CI***	***n***	***%***	***95% CI***	***n***	***%***	***95% CI***	**F value**	**p-value**
**Nationality** South African citizen Other SADC Other African None	1,181 9 2 2	98.9 0.8 0.2 0.2	97.8–99.5 0.3–1.8 0.04–0.7 0.04–0.7	550 0 0 1	99.8 0 0 0.2	98.5–100 - - 0.02–1.5	631 9 2 1	98.1 1.4 0.3 0.2	96.1–99.1 0.5–3.8 0.1–1.3 0.02–1.3	1.9	0.16
**Facility type** Clinic Gateway Hospital Mobile clinic	798 220 101 75	66.8 18.4 8.5 6.3	53.8–77.7 12.0-27.3 2.1–28.2 3.1–12.4	358 101 57 35	65.0 18.3 10.3 6.4	42.1–82.6 6.4–42.4 2.1–38.3 2.6–14.6	440 119 44 40	68.4 18.5 6.8 6.2	53.4–80.4 10.3–31.0 2.1–20.0 3.1–12.0	0.2	0.78
**Sex** Female Male	847 347	70.9 29.1	67.7–74.0 26.3–32.0	419 132	76.0 24.0	71.0-80.4 19.6–29.0	428 215	66.6 33.4	62.5–70.4 29.6–37.5	8.5	**0.01**
**Ethnicity** Black African Colored/Mixed ancestry Indian/Asian	1,191 2 1	99.7 0.2 0.1	99.2–99.9 0.04–0.6 0.01–0.7	551 0 0	100 0 0	- - -	640 2 1	99.5 0.3 0.2	98.6–99.9 0.1–1.1 0.02–1.5	1.4	0.28
**Education level** No education Primary education High school education Tertiary education	21/1,178 60/1,178 678/1,178 419/1,178	1.8 5.1 57.6 35.6	0.8–3.9 3.9–6.7 52.1–62.8 29.3–42.4	5 25 303 215	0.9 4.6 55.3 39.2	0.4-2.0 2.7–7.6 48.0-62.4 30.9–48.2	16 35 375 204	2.5 5.6 59.5 32.4	1.1–5.9 4.3–7.2 54.5–64.4 27.3–37.9	2.9	**0.05**
**Age, median (IQR)**	1,193	30	25–37	551	30	26–37	642	30	25.0–37.0	-1.5*	0.14
**Age categories** 18–24 years 25–29 years 30–34 years 35–49 years 50 + years	258/1,193 305/1,193 250/1,193 318/1,193 62/1,193	21.6 25.6 21.0 26.7 5.2	18.4–25.3 22.3–29.1 18.4–23.8 23.3–30.3 3.9-7.0	112 136 121 156 26	20.3 24.7 22.0 28.3 4.7	15.3–26.6 21.0-28.8 17.9–26.7 24.1–32.9 3.3–6.7	146 169 129 162 36	22.7 26.3 20.1 25.2 5.6	20.1–25.6 21.4–31.9 17.1–23.5 21.0–30.0 3.7–8.4	0.7	0.56
**Marital status** Married (living together) Married (living separately) Cohabiting Dating Single	73/1,169 32/1,169 169/1,169 727/1,169 168/1,169	6.2 2.7 14.5 62.2 14.4	3.4–11.3 1.7–4.3 10.5–19.7 56.8–67.3 9.6–21.1	27 11 83 336 88	5.0 2.0 15.2 61.7 16.1	3.4–7.1 1.0–4.0 10.5–21.5 55.0-67.9 9.5–26.1	46 21 86 391 80	7.4 3.4 13.8 62.7 12.8	3.0-16.9 2.1–5.4 9.3–20.0 55.7–69.2 8.6–18.7	0.9	0.46
**Access to US$14 in emergencies** Very/somewhat difficult Fairly/very easy	703/1,173 470/1,173	59.9 40.1	49.6–69.4 30.6–50.4	348 198	63.7 36.3	55.9–70.9 29.1–44.1	355 272	56.6 43.4	41.1–71.0 29.0–59.0	1.0	0.34
**Received child support grant**	531/1,169	45.4	42.4–48.5	269	50.7	44.9–53.3	262	42.2	37.9–46.6	5.8	**0.03**
**Area of residence** Rural Urban	441 753	36.9 63.1	16.1–64.1 35.9–83.9	144 407	26.1 73.9	8.3–58.0 42.0-91.7	297 346	46.2 53.8	20.2–74.5 25.5–79.9	2.0	0.18
**Mode of transport** Foot Public transport Private transport Other	492/1,179 611/1,179 70/1,179 6/1,179	(51.3%) 51.8 5.9 0.5	26.2–59.1 35.7–67.6 3.8–9.1 0.2–1.2	206 309 30 3	37.6 56.4 5.5 0.5	19.4–60.1 35.6–75.2 3.1–9.4 0.2–1.6	286 302 40 3	` 45.3 47.9 6.3 0.5	30.7–60.8 32.7–63.4 3.7–10.6 0.1–1.8	1.0	0.38
**Time to get to facility** < 30min 30–60min > 60min	705/1,172 405/1,172 62/1,172	60.2 34.6 5.3	47.5–71.6 23.5–47.6 3.7–7.6	366 159 22	66.9 29.1 4.0	58.6–74.3 22.1–37.3 2.5–6.4	339 246 40	54.2 39.4 6.4	37.0-70.5 22.9–58.6 3.8–10.5	2.0	0.18

p-value of$\le 0.05$ was considered statistically significant

p-values derived using Mann Whitney U-test for continuous data

p-values derived using Chi-squared test and considered the one stage cluster design

proportions (%) for the columns reported as n/N (except if data is missing - denominator added in the n column) and the associated 95% CI

IQR – Interquartile range; CI – Confidence Interval; US$ – United States of American Dollar; RiC – Retained in Care; nRiC – Not retained in care

* z value reported

Over a third (75.9% − 418/551) of those retained in care at 12 months were virally suppressed based on the records from the NHLS database. The mortality in this HIV-positive cohort at 12 months was 5.2% (62/1,194). A higher proportion of those who died were female (54.8%, 34/62); however, we could not ascertain the cause of death from the RMS.

## Discussion

In this study, we sought to investigate the proportion of PLHIV linked to care within 3 months after testing HIV-positive and those retained in care 12 months after initiating ART in a rural district in KwaZulu-Natal (KZN), South Africa. Our study recorded a linkage to care rate of 83% in the first three months after testing HIV-positive, which is higher than findings from other studies. Johnson et al. [22] reported a national linkage rate of 57% and 62% for KZN. However, the Human Sciences Research Council (HSRC) study reported a linkage rate of 76% for the uThukela DM [4]. There is scarcity of reports on the progress made on both linkages to care and ART initiation since the implementation of UTT and same-day initiation. Variations in the definitions and time points for measurements from HIV-positive diagnosis to ART initiation also make it difficult to compare results obtained from different studies. A study conducted in South Africa to assess the impact of a health app for Android smartphones providing HIV-related laboratory results, information, support, and appointment reminders to engage and link patients to care indicated that before the intervention, only 47% (162/345) of the study participants were linked to care between two weeks and eight months [23]. This was before UTT was adopted in South Africa in September of 2016, which could explain the lower linkage to care rates compared to our study findings obtained from data collected between 2017 and 2019.

Although three months from date of diagnosis was the time frame used to define linkage to care before UTT, Rosen, and colleagues [24] argue that in the context of UTT where treatment uptake is meant to be accelerated, 28 days from date of diagnosis has demonstrated to be a more appropriate time frame to measure linkage to care. A recent priority setting meeting for HIV service delivery research and guidance consultation held at the end of 2018 organised by the World Health Organization (WHO) suggested that rapid ART initiation should consider ART initiation within 7 days with the offer of same-day ART. It is argued that reductions in the time frame for measuring linkage to care, to 7 days and 28 days from date of diagnosis, would provide a better reflection of the impact of UTT on the rate of ART uptake. The high linkage to care rate (83%) observed in the study may have been related to the fact that we reported on linkage to care at 3 months from date of diagnosis, as the UTT initiation protocols had not yet been fully adopted in all the provinces and facilities in South Africa. However, our study shows that 61% of the HIV-positive participants linked to care within 14 days of being diagnosed HIV-positive. Even when same day ART initiation is available for all people who test positive for HIV, psychosocial and health systems factors have contributed to delays for many patients in initiating ART [25, 26]. Without appropriate linkage to care interventions, UTT is unlikely to be successful [27], especially because through UTT more people become eligible for ART [11].

An eight-year longitudinal study conducted before the UTT era in KZN, South Africa identified linkage to care *(*defined as *“the proportion of patients who engage with formal health-care sector for HIV-related health care”)* as the biggest weakness of treatment programs, with less than half (45%) of the 82% PLHIV who knew their HIV status successfully linked to care [8]. Within the UTT era, our study indicated improved linkage to care rates (83%) with very poor rates of overall early retention in care at 12 months. This finding implies that efforts towards improving retention and viral load suppression among PLHIV should be strengthened through the adoption of differentiated service delivery models such as the Fast-Track Treatment Initiation Counselling [28]. Although our study did not indicate any gains in early retention in care in the era of UTT, gains of UTT on early linkage to care is confirmed in another study conducted in Malawi [29]. Alhaj and colleagues [29] found that PLHIV initiated under the UTT era showed increased early retention in care behaviors compared to those initiated before UTT (83.0% compared to 76.2%, respectively).

In our study, a higher proportion of men were linked to care compared to women within three months. Another study conducted in the provinces of Gauteng and Limpopo of South Africa also showed comparative linkage to care rates for men and women up to 90 days but became lower in men during the 90–365 days after testing [30]. While we found that a greater proportion of men were becoming aware of their HIV status and were linked to care compared to women, women demonstrated better treatment-seeking behaviors. Several studies across the sub-Saharan continent have confirmed that women have better retention in care behaviors [22, 31]. For instance, a multi-center study conducted in South Africa showed that men were more likely to be lost to follow up compared to women [32]. The reason for the gender differences has been attributed to masculinity – a set of local beliefs and practices that capture what it means to be a man in a particular context [33]. In South Africa, gender-transformative interventions such as “One Man Can”, a rights-based gender equality and health program intervention, and Decentralized Medication Delivery (DMD) have shown success in reducing masculinity-related barriers to engaging in HIV services [34]. A recent study conducted in South Africa revealed that these differentiated service delivery models have the potential to increase the retention to care and adherence to medication among men in particular [35]. These models achieve this by helping men refashion ART-friendly masculinities – a set of attributes, behaviors, and roles associated with boys and men that favor the uptake and use of ART [36]. Other interventions/ models designed to help South African men initiate ART and remain in care include the MINA and Coach Mpilo campaigns, which provide men with information and support that help them to get tested for HIV, to initiate and remain in care [37].

Considering the geographic distance for participants located in the rural areas accessing HIV testing services in facilities, and the cost of public transport for the majority who used facilities in urban areas, it was not surprising to find issues of lack of transport to health facilities emerging as a barrier to retention in care in this context. Our study found that participants who used public transport to access health facilities were more likely to be in care after 12 months than those who access health facilities by foot. Similar findings on access to health facilities have been noted in other low- and middle- income countries [38, 39]. Unlike studies conducted in urban settings, our study demonstrated that HIV patients in uThukela District Municipality do not often change health facilities. Although participants remained in their testing facilities and reported feeling that they were provided with necessary information and generally treated well by clinic staff, some felt that clinical personnel did not have enough time for them when they visited the health facilities. This might have also contributed to the reasons for not remaining in care. Lankowski et al. [38], identified the size of the facility and staffing-patient ratio as factors that may improve linkage and retention in care. This calls for recruitment of more HIV counsellors and linkage officers in uThukela health facilities.

Other reasons for delaying the return to the clinic for HIV care or non-retention in care included inability to take time off work or inconvenient appointment dates. Our findings corroborate the study by Govindasamy et al. [40], which showed an association between being employed and being less likely to be linked to care, perhaps due to the difficulties of accessing health care services after working hours.

Our study found a statistically significant difference between participants who remained in care at 12 months and those who dropped out of care for several variables. Access to cash was significantly associated with retention in care. Our findings show that participants who had no access to a child support grant and those who found it very difficult to access R200 ($12) in emergency cases were less likely to be retained in care at 12 months post ART initiation. This corroborates findings from a similar study [41] where personnel responsible for linking clients to care had to provide transport for clients who did not have enough money to go to the health facility.

Participants’ sex and age played a major role in determining whether they test for HIV, link to, or remain in care. The proportion of women that tested for HIV and retained in care at 12 months in this study was significantly higher than men. This corroborates studies that show that HIV-positive men are less likely than women to access HIV care [42, 43], and be retained in care across sub-Saharan Africa [44]. However, a higher proportion of males were linked to care within three months post HIV-positive diagnosis compared to females in our study. More research is needed to understand barriers to early linkage and retention for men. We also found that females in the younger age and males in the older age categories had higher rates of positivity. This was consistent with the current research done in KZN through the Centre for the AIDS Program of Research in South Africa (CAPRISA).

## Strengths and limitations

We found the availability of a medical clinic visit evidenced by a record in Tier.Net, which was synonymous with ART initiation, to be useful in determining linkage to care. However, in some instances, after receiving counseling and blood samples drawn at a treatment facility, the individual might indicate that they are not ready to initiate treatment even though there was UTT.

Our determination of retention in care was confirmed via the presence of viral load measurements and the confirmation of deceased status with the Department of Home Affairs. Identifying viral loads and the subsequent viral suppression after one year of linkage to care through the NHLS is superior to other methods such as pharmacy refills and clinic attendance records reported as retention in care because viral load measurements capture PLHIV who had transferred out from one facility to another.

This cohort study has provided an overview of the demographics and context of the participants, and it shows a high linkage rate as evidence by ART initiation in the context of UTT amongst the HIV-positive cohort. This could have been influenced by our field team, who provided educational materials about linkages and support to the participants.

This study is of great relevance within the context of the South African health priorities, and the findings will provide insight to guide decision makers, especially the National Department of Health (NDoH) on ways to strengthen strategies geared towards improving linkage to and retention in HIV care. Although the study focused on one district with a high HIV prevalence, most of the findings maybe applicable to other settings.

A limitation to our study though is that we did not investigate the presence of a CD4 count, which is a good tracker for each time the participant interacts with the health system. The study also, did not capture the details of interventions that are being implemented in our study sites to show which interventions enhanced linkage to care.

## Conclusion

This study has shown higher rates of linkage to care compared to regional estimates, but less than 50% of the participants remained in care at 12 months, pointing to challenges with access to continuing care. We have also described socio-demographic characteristics, and some drivers of accessing care such as access to cash, lack of transportation to health facilities, gender, and age.

Young women test more for HIV compared to young men. This may be attributed to the fact that they have more opportunities for testing when accessing family planning or maternal and child health (MCH) services. While integrated family planning or MCH services may have improved testing for women of reproductive age, there is a need to improve testing for older women and younger men. Our findings suggest that interventions could target men aged 18–34 years to encourage HIV testing and linkage to care. More research is needed to understand barriers to care linkage and retention for men.

## Data Availability

The datasets generated and/or analyzed during the current study are available from the corresponding author on reasonable request.
